# Mr. Hicks, on Hydrophobia

**Published:** 1807-03

**Authors:** George Hicks

**Affiliations:** Baldock


### *To the Editors of the Medical and Physical Journal.*

Gentlemen,

Among the many unfortunate cases which have happened from the bite of Mad Dogs, the following has fallen under my care, and as it possibly may throw some light on the treatment of a disease for which at present we have no specific, by inserting it in your useful and beneficial Journal, you will very much oblige,

Gentlemen,

Yours, respectfully,

*Baldock, Feb.* 14, 1807. GEORGE HICKS.

---

On Sunday evening, Nov. 30, 1806, I received a note from the Rev. Dr. Heath, of Walkern, Herts, informing me, that Wm. Miles, a young man of his parish, was taken on that day in a frantic state, and which, it was supposed, proceeded from the bite of a mad dog, and wished me to ride over and see him. I at the time being particularly engaged, sent my apprentice, and directed him, if he found him much affected with spasms about the præcordia and fauces, to give him a fourth part of the following mixture, every two hours.

Tr. Asafœtidæ ℥j. Mist. Camphorat. ℥vij. Spt. Ammon. C. ʒiv. Tr. Opii ʒij. Misce ft. mixt.

On Monday, December 1st, I saw him, and found him in a cottage, on a bundle of straw, handcuffed, with a cart rope passed several times round his body, fastened to strong stakes, driven into the ground floor for that purpose, in such a way, that he could move no other part but his head. Upon asking those who attended him, what induced them to bind him in the way they had done, they said, he had been bit by a mad dog, and that the disease had taken place: that on the Sunday afternoon he came into his mother's house, (who lives at Walkern,) at the back door, and passed immediately through to the front door, where he stopt for the space of two minutes; he appeared much agitated, and as she says, his throat projected out almost to his chin; he grated his teeth very much, and appeared incapable of swallowing his saliva, which he continually attempted to do. His mother asked him to drink some beer; he said, Drink! no! I have been bit by a mad dog, and immediately left the house. At a small distance from the house, he met too men, both of whom he shook and bit. Soon after, he passed out of the road through a thick hedge into a close, which he crossed, and on the other side of the close, being stopped by some strong oak railing, he grasped it, and violently shaking it, broke it. He then went into a farm yard, and laid himself down in the corner of a cart hovel; he could not lie long, but soon began to roll about. His mother having followed followed him, looked into the cart hovel; he begged her to keep from him, as from his distressed feelings, he could not answer for what he did, upon which she ran away.— He soon came out of the cart hovel, and crossing the farm yard, attacked a bullock, after which he went towards the house, which is at some distance from the farm yard. In the porch of the house was lying a large, savage, mastiff dog; the dog came out of the porch at him, but before it approached half way towards him, it turned back, and ran into the porch; he followed the dog, attacked it, got it down, and from his attitude, a young man was looking on, supposes he attempted to bite it; the dog got from under him and ran away. He then went into the field, a number of men followed him, caught him, handcuffed him, brought him to Walkern, and bound him in the manner I saw him.

I then asked Miles how he felt himself; he said, he felt pain in the part of his leg the dog had bit, which extended up the inner side of his thigh to the pit of his stomach, and which felt to him as tight as if a strong cord was bound round him, from that part, up to his throat, (which appeared swollen) with great difficulty in swallowing.

I desired those who had the care of him, to unbind him, that he might point out to me the part that had been bit. Upon uncovering his legs, he complained of the coldness of the air, (alth ugh from the number of people present, it was much heated,) and begged that they might be covered up again, as he could not bear it, saying, it increased the tightness of his stomach and throat. On examining his legs, I perceived the cicatrix of a small wound on the outer side of his right leg, about two inches below the superior end of the fibula, in a state of inflammation, very similar to the pustule from inoculation of the small-pox, about the eighth day, excepting its containing little or no fluid, and being more inflamed and elevated. I observed to those present, that when they raised him up, I expected he would point out the inflamed cicatrix, as the part the dog had bit, which he did, and upon touching it with his finger, he immediately complained of the tightness of his stomach and throat being increased. This induced me to touch the part two or three times with my finger, and the spasmodic affection of the præcordia and fauces became more or less increased, according to the degree of pressure on the part. I then asked him where he was when the dog bit him, and how it came to bite him. He said, he was bit in the field, as he was looking for his master's master's horse, by a lurcher dog; he did not see the dog till after it had bitten him; it bit him as it passed by him, and went trotting on; soon after which, two men came into the field, and asked him, if he had seen a lurcher dog; he said, yes, it had bitten him, and was gone on before. The men informed him the dog was mad, and that they followed it to shoot it, one of them having a gun for that purpose. I could not then get him to say how long it was since he was bit, for though he appeared in every respect reasonable, yet from the spasmodic affection of the fauces, &c. he could not converse much. Upon asking him, if he had a wish to be unbound, he said, Yes, I should like to be unbound, that I might go into the field. I informed him, that if he was unbound, he must stop with the men who had the care of him; he said, he could not stop, but must go. I asked him, where? he said, he knew not where, but must go into the field; I then said, you must remain where you are.

The spasmodic affection of the præcordia and fauces being evidently increased by the slightest pressure on the inflamed cicatrix, I was induced to apply a caustic to it, about two inches in length, and nearly the same in breadth, hoping, that if the spasmodic affection of the præcordia and fauces proceeded from nervous sympathy, that by destroying the nerves of the part, the nervous system might be relieved, and the spasmodic affection depending thereon, removed. At the same time, I directed the antispasmodic mixture to be continued as before, and made use of mercurial friction to the thigh.

From a number of engagements, it was not in my power to see him again earlier than Wednesday evening, December 3, when I found him much the same, except the spasmodic affection of the præcordia, &c. being increased. Upon examining his leg, I found the caustic from some cause or other had not had the desired effect, having acted but partially. I therefore applied another of the same size, and not only secured it with adhesive plaster, as I had done the former, but bound it on with bandage, to prevent those about him from disturbing it. Prior to my applying the second caustic, I made a slight pressure with my finger on the cicatrix, and found it increased the spasmodic affection of the præcordia, &c. as on the Monday.

I called on him again on Friday evening, December 5, about nine o'clock, and learned from those who attended him, that the spasmodic affection of his throat, &c. became much increased on the preceding day, (Thursday.)

so much so, that he appeared greatly agitated at the sight of water, and would not suffer it to approach his lips, throwing his head violently back, when it was brought to him, saying, it terrified him. He expressed the same aversion to water on Friday evening, at the time I was with him, and which had continued from the Thursday.

On the Sunday, Monday, Tuesday, and Wednesday, he did not appear to have a greater aversion to water than to other fluids. From the first he swallowed fluids with difficulty, more so at some times than at others, as the spasms were more or less violent. On examining his leg, I found the caustic that was applied on the Wednesday, had had the desired effect, and produced a large and good eschar; upon touching which, he complained of the spasmodic affection of the præcordia, &c. being increased. To use his own words, by touching the part, the cords or ropes with which he felt as if he was bound, were tightened. This induced me to apply a third caustic, much larger than either of the other, about four inches long, and three wide; I likewise applied it very thick, with a view of its penetrating deeper than the others had done; it gave him great pain for several hours.

On Saturday morning, December the 6th, about nine or ten o'clock, he informed his attendants that he was much relieved, that the tightness from the pit of his stomach to his throat was removed, as if the cords he had been bound with, were cut; his aversion to water left him, he swallowed with ease; the terror and agitation of his mind going off, he became calm and quiet.

I called upon him again on Sunday, December the 7th, and was much pleased to find this alteration; he appeared perfectly himself, not having the least spasm about him. Upon asking him if he could call to mind the day on which the dog bit him, and in what way the wound went on, he informed me, that it was on the Wednesday week before he was taken ill, which was November the 19th; that after he got home with his master's horse, his leg smarting, he put down his stocking to look at the part; it had bled a drop of blood, which coagulating stuck his stocking to it. As the place was but small, he did nothing to it, nor said any thing about it. It healed up, as he supposed well, with a small dry scab on it, which, in five or six days was rubbed off. It continued to look well till the Thursday week, November the 27th, when it itched and appeared rather red.

On

On the Friday, November 28th, he felt slight prickling pains in it, and the redness increased.

On Saturday, November the 29th, the pain in the part increasing, about noon his knee became stiff, and in the course of the afternoon the stiffness with some pain extended up the innerside of his thigh. In the evening, about nine or ten o'clock, he was taken with great coldness, so much so, that he crept down into his bed so as to cover his head with the bed clothes, and, as he expressed himself, he could not bear the cold wind to come to his face.

On the Sunday morning, as soon as he was up, the sensation of coldness increasing, he went to the fire, and in eating his breakfast, found his throat stiff, and swallowed with difficulty. Soon after breakfast he left his master's house, in what way he knows not, feeling himself hurried. He recollects falling down in a field, and on getting up, was taken with pain aud stiffness at the pit of his stomach, which extended up to his throat, as if he had been bound with a strong cord. He now became more hurried, and knew not where he went, having, from his distrest feelings, a very imperfect idea of what followed, till after he was secured, when his reason returned.

Upon being asked why he disliked water, and would not suffer it to come to his mouth, he said, as soon as he saw it, his eyes seemed to turn round in his head, he became giddy and much terrified, and on being asked, why he should have gone into the field,' had he been unbound, he said, from the terror he had about him, it would have been impossible for him to have kept himself still.

I think it necessary to observe, that the antispasmodic mixture was taken regularly, till he found himself better, and that mercurial friction was made use of every night, yet from the great change that took place so soon after the application of the third caustic, together with the nervous communication there appeared to be between the cicatrix and the præcordia and fauces, I am strongly of opinion, that the caustic had the principal share in effecting the cure, by destroying the extremities of the nerves that were thus excited by the morbid action that was going on in the cicatrix.

It is well known, that the virus of the mad dog, when applied to a wound, remains for a time local, and which time, unfortunately, is not determined as in variolous and vaccine inoculation; but whether it remains local one month or twelve, the cicatrix of the wound is always ininflamed inflamed prior to the system's being affected, and it is allowed by all practitioners, that removing the part during the local state of the virus, either by excision or caustic, will effectually prevent its affecting the system, and indeed, it is the only remedy that can be depended on. But after the cicatrix has become inflamed, and the system affected, supposing absorption to have taken place, the application of a caustic is not thought of; and was the system affected in this way, it is impossible it could have the least effect in relieving it. But then, if the virus of the mad dog was to pass the lymphatic system, and be conveyed into the arterial system, would it not produce an excitement thereon; that is, would it not in the same manner as the absorption of variolous, or vaccine virus, or that of common pus, increase the action of the heart and arteries. In Miles's case, his pulse was not increased, it remained perfectly natural, neither had he the goose-skin appearance which accompanies rigor produced by absorption, nor the increased heat on the surface that follows. His skin was smooth; heat natural; and though he complained of great coldness when uncovered, it appears to have arisen from the stimulus of the external air acting on the nerves of the skin, which were too irritable to bear its impulse, and which is peculiar to the disease.

As hydrophobia seems to be a disease of the nervous system, and that the spasmodic affection of the præcordia, &c. produced thereby, destroys the patient, may not the virus first act on the nerves of the part, and from thence the nervous system become affected, independant of absorption, as in trismus and tetanus, where the inflammation of a nerve produced by puncture, or contusion, &c. has not only occasioned rigidity of the muscles of the face and neck, but even of the whole body, and yet no one supposes this rigidity is produced by absorption; and as the division of a nerve or nerves thus affected, has relieved the nervous system, and the spasmodic affection of the muscles, depending thereon, has, in a short time, been entirely removed, may not the solution of the extremities of the nerves that are acted upon by the virus of the mad dog, relieve the nervous system in the same way?

I was called to Mr. Wm. Moulden, cooper, of Stevenage, (who is now living and in good health) on the 10th of October, 1797; he was then affected with trismus, his jaws had been compleatly locked for four days, during which time he had taken no other nutriment than thin gruel, gruel, which he sucked through his teeth. On enquiry I learned, that on the 14th of September he had the misfortune (in making a butt) to bruise the palm of his left hand; he continued to work at his business for four or five days, when a slight inflammation in the bruised part came on, accompanied with a good deal of pain; soon after which, his cheeks and fauces became stiff, which stiffness daily increased, until his jaws became locked. In the palm of his hand there was a small tumour, situated between the metacarpal bones of the fore and middle fingers, in a state of suppuration; I proposed making an incision through the whole tumour, cutting down between the metacarpal bones, with a view of relieving the nervous system by a division of the nerves affected; which being assented to, I accordingly did, and wrapt his hand up in a large saturnine poultice, with two ounces of tincture of opium in it, which was repeated every six hours. I likewise directed him to take three grains of purified opium every two hours. The next day I had the pleasure to find he could open his mouth, and swallow fluids with ease, the rigidity of the muscles of his face and throat being in a great measure removed. The wound soon healed, and his hand became, in every respect, as well as before he received the injury. Trismus, in this case, being removed so soon after the operation, I attribute more to the division of the nerves affected, than to the effect of the opium.

In giving Miles's case, with the remarks and observations thereon, it is very far from my wish to assume a discovery; but from the manner in which he was relieved, apparently by the caustic, I feel it my duty to communicate it to the public, at the same time, earnestly requesting those, who may be called to patients labouring under this most distressing of all diseases, to give it a trial, at least with other remedies; and if it should be found to succeed, I shall feel myself amply rewarded.

N. B. The eschar produced by the caustic exfoliated very kindly, leaving a healthy wound, which incarnated and cicatrized very fast; he is in good health, and has returned to his usual employ, that of a farmer's servant.

